# Characterization of metabolic differences between embryogenic and non-embryogenic cells in forest trees

**DOI:** 10.1186/1753-6561-5-S7-P146

**Published:** 2011-09-13

**Authors:** So-Young Park, Wi-Young Lee, Yong-Wook Kim, Heung-Kyu Moon

**Affiliations:** 1 Korea Forest Research Institute

## 

**Figure 1:**
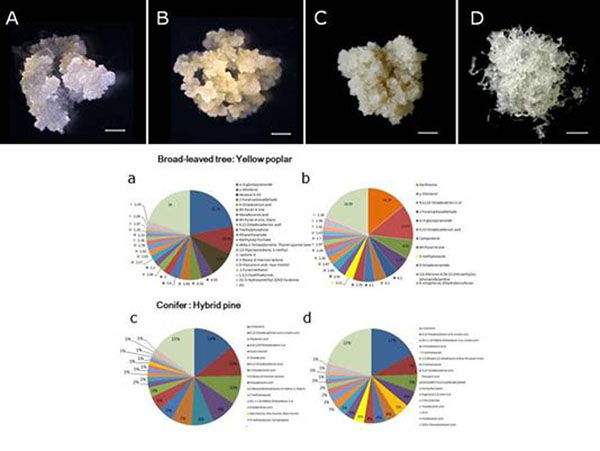
Non-embryogenic (NEC; A, C) and embryogenic cells (EC; B, D) of yellow poplar and hybrid pine ‘rigida x taeda’ respectively, and its metabolic compositions (NEC: a, c and EC: b, d).

Somatic embryogenesis (SE) in forest trees is considered as one powerful approach in cloning elite genotypes. However, SE is difficult to achieve in tissue or cell beyond the mature embryo phase in trees and its physiological process involved are also poorly understood. Recently metabolic profiling studies have been contributed to understand the mechanism involved in SE process of plant [1-3]. In addition, there are also a number of reports indicating that polyamines (PAs) play a crucial role in SE process [4,5]. As part of an ongoing study of the transition of somatic cells to an embryogenic state in adult trees, embryogenic (EC) and non-embryogenic cells (NEC) in various forest species were investigated for its metabolic compositions including PAs. A comparison of metabolic compositions of NEC and EC using gas chromatography/mass spectrometry (GC/MS) identified around 50 compounds, partly displaying significant changes in metabolite levels, e.g., highly elevated levels of xanthosine and methyloxazole in EC compared to NEC of broadleaves and conifer species (Fig.1). Changes in the polyamine content were also analyzed in both cell types. Free polyamine contents varied according to the species and cell types, the highest levels occurring in the NEC on proliferation medium, when putrescine and spermidine were most abundant. However, the putrescine/spermine+spermidine (put/spm+spd) ratio was higher in EC of yellow poplar and hybrid pine. Analysis of PAs in both cell types indicated that total polyamine concentration was always higher in NEC than in EC, and spermine was present in only minute quantities and showed only a small change. From this analysis, we have identified numerous compounds involved with embryogenic state, and could characterize its differences between broadleaves and conifer trees.
